# Self-care practice and its predictors among adults with diabetes mellitus on follow up at public hospitals of Arsi zone, southeast Ethiopia

**DOI:** 10.1186/s13104-021-05511-0

**Published:** 2021-03-19

**Authors:** Rahel Nega Kassa, Ibrahim Yimer Ibrahim, Hana Abera Hailemariam, Mekdes Hailegebriel Habte

**Affiliations:** 1 Department of Surgical Nursing, Saint Paul Hospital Millennium Medical College, Addis Ababa, Ethiopia; 2 Ethiopian Midwives Association, Addis Ababa, Ethiopia; 3 Department of Neonatal Health Nursing, Saint Paul Hospital Millennium Medical College, Addis Ababa, Ethiopia; 4 Department of Pediatrics Nursing, Saint Paul Hospital Millennium Medical College, Addis Ababa, Ethiopia

**Keywords:** Diabetes mellitus, Self-care practice, Hospital, Arsi zone, Ethiopia

## Abstract

**Objective:**

Diabetes mellitus is a chronic illness that requires ongoing patient self-management and support to prevent acute complications and to reduce the risk of long- term complications. The objective of the study was to assess diabetic self-care practice and its predictors among adults with diabetes mellitus on follow up at hospitals of Arsi zone, southeast Ethiopia.

**Results:**

Above half (53.3%) of diabetic patients had good self-care practice. Younger age (AOR: 8.95, 95% CI 1.89,42.48), earning a high income (AOR: 2.495, 95% CI 1.0,5.85), having a family history of diabetes (AOR: 4.5, 95% CI 1.3, 15.5), long duration since the diagnosis of diabetes (AOR: 2.14,95% CI 1.127,4.05), not having diabetic complications (AOR: 3.87, 95% CI 2, 7.48), and having glucometer (AOR: 4.08, 95% CI 1.78 l, 9.33) were significantly associated factors with good diabetic self care practice. Efforts should be made to prevent complications of diabetes mellitus, to support patients who are aged and who have no glucometer at their home to promote good self-care practice. Particularly, health care providers should give special attention to newly diagnosed patients.

## Introduction

Diabetes mellitus (DM) is a chronic illness that requires continuing medical care and ongoing patient self-management, education, and support to prevent acute complications and to reduce the risk of long-term complications [1–5].

International Diabetes Federation (IDF) estimated that the global diabetes prevalence in 2019 to be 9.3% (463 million people), rising to 10.2% (578 million) by 2030 and 10.9% (700 million) by 2045 [6]. Additionally, WHO estimated DM causes 3.2 million deaths per year, six deaths every minute and 8700 deaths every day [7].

A systematically reviewed study conducted in Ethiopia showed that the prevalence of diabetes in most localities of Ethiopia appears to have increased from that estimated by the IDF in 2015 that was 3.9% [8]. Another study conducted in Ethiopia showed that almost half (49%) of the participants had long term diabetic complication confirmed medically [9].

In contrast, patients who have adequate self-management have better outcomes, live longer, enjoy a higher quality of life, and suffer fewer symptoms and minimal complications [10–12]. That means inadequate diabetic self-management is associated with higher patient morbidity and mortality as well as increases the costs of medication and laboratory tests and cost in time and effort of the care providers [13].

Though several related studies were conducted in different parts of Ethiopia and identified few factors associated with diabetes self-care practice, there is no study conducted on this topic in the present study area. As a result, this study was intended to assess the diabetic self-care practice and its predictors among adults with DM on follow up at hospitals in Arsi zone, southeast Ethiopia; The finding of the study shows the degree of the problem and contribute to bridging information gap so as to help improve the health care delivery system in terms of enhancing the diabetic self-care practice.

## Main text

### Study area and period

The study was conducted from April 12, to May 12, 2017at four public Hospitals (Asella referral Hospital, Arsi Robe Hospital, Abomsa Hospital and Bekoji Hospital) in Arsi zone, South East Ethiopia.

### Study design

A cross-sectional study design was employed.

### Study population

All sampled adult patients with diabetes who were visiting public Hospitals in Arsi zone at diabetic follow up clinic.

### Eligibility criteria

Patients with diabetes aged 18 years and above who had regular follow-up for at least 6 months. Patients who were not mentally and physically capable of being interviewed were excluded.

### Sample size determination

The study used single population proportion sample size determination formula. The prevalence rate of good diabetic self-care practice was taken as 76.8% [12] with the assumption of 95% confidence interval (CI) and 5% marginal error. Adding 10% contingency for possible non-respondents, the final total sample size became 301.

### Sampling technique

First, the monthly flow of patients in each hospital was determined by using the patients’ appointment list. The total number of patients who had a follow-up in those four hospitals was 1502 [14]. Then, every fifth patient was selected for interview by applying a systematic random sampling technique. Finally, a lottery method was used to select the first study participants.

### Data collection techniques and instrument

Data were collected by using a structured questionnaire through a face-to-face interview. Clinical data about diabetes complications and comorbidities were extracted from each patient's medical chart. A Fasting Blood Sugar (FBS) test was performed for all study participants during data collection time. Eight nurses were involved in the data collection process after they had been trained about the objective of the study and how to collect data. Afan Oromo language version of the questionnaire was used for the data collection. The quality of the data was assured by using a validated questionnaire, translation, and retranslation of the questionnaire. The questionnaire was translated from the English language to Afan Oromo and back to English by another translator. Besides, the questionnaire was pretested on 15 patients with diabetes who were 5% of the total sample size, and necessary adjustments were made before its use for actual data collection.

The data collection tool incorporated three parts. Part I: includes socio-demographic characteristics, Part II: encompasses the clinical characteristics of the respondents, and Part III: consisted of the diabetic self-care domains which were adapted from Summary of Diabetes Self-Care Activities (SDSCA) [15].

### Data analysis

Data were cleaned, coded, and entered into Epi info version 7 and analyzed using SPSS version 21. Descriptive statistics were computed to describe the sociodemographic and clinical characteristics of the study participants. Binary and multivariable logistic regression analysis were performed to examine the existence of a relationship between diabetic self-care practice and the independent variables listed below. A p value of < 0.05 on a binary logistic regression was considered to select candidate variables for multivariable logistic regression analysis as well as to declare a statistical significance. The results were presented in the form of text and tables.

### Study variables

#### Dependent variables

Diabetic self-care practice.

#### Independent variables

Socio-demographic factors: Sex, age, marital status, educational status, religion, and monthly income.

Clinical characteristic: type of DM, type of treatment, duration of DM diagnosis, co-morbidity, and presence of complications.

### Operational definition

#### Self-care practice

Study participants who scored equal to or above the mean in the SDSCA were classified as having good diabetes self-care practice and those who scored below the mean were considered as having poor self-care practice.

## Results

### Socio-demographic characteristics of respondents

Out of 301 study participants planned for the study, 299 of them were enrolled in the study yielding a response rate of 99.3%. The mean (± standard deviation) age of respondents was 39 ± 15.8. Most (44.8%) of the study participants attended college and above (Table 1). About half (52.8%) of the respondents earned low monthly income whereas 67.3% of them were married.

**Table 1:** Socio-demographic characteristics of diabetic patients on followup at public hospitals of Arsi zone, southeast Ethiopia, 2017 (n = 299)

Variables	Category	Frequency	Percent
Sex	Male	183	61.2
	Female	116	38.8
Age	18–24	63	21.1
	25–34	70	23.4
	35–44	56	18.7
	45–54	48	16.1
	55–64	37	12.4
	> 65	25	8.4
Religion	Muslim	142	47.5
	Orthodox christian	132	44.1
	Protestant	22	7.4
	Catholic	3	1.0
Educational status	Cannot read and write	65	21.7
	Can read and write	38	12.8
	Elementary	41	13.7
	High school	21	7.0
	College and above	134	44.8
Marital status	Single	86	28.7
	Married	202	67.3
	Divorced	7	2.3
	Widowed	4	1.7
Monthly income	Low	158	52.8
	Medium	42	14.0
	High	99	33.1
Occupation	Farmer	162	54.0
	Private business	55	18.3
	Student	46	15.3
	Employed	36	12.0
Place of residence	Urban	130	43.3
	Rural	169	56.7

### Clinical characteristics of respondents

Majority (58.2%) of the study participants had type 1 diabetes. Most (66.2%) of the respondents had normal body weight (18.5–24.9 kg/m^2^). A large proportion (86.9%) of the respondents did not have a glucometer. The vast majourity (74.9%) of the study participants had not experianced diabetic complications and 72.9% of them had no comorbidities (Table 2).

**Table 2:** Clinical characteristics of diabetic patients on follow-up at public hospitals of Arsi zone, southeast Ethiopia, 2017 (n = 299)

Variables	Category	Frequency	Percent
Type of DM	Type 1	174	58.2
	Type 2	125	41.8
Family history	Yes	61	20.4
	No	238	79.6
Type of treatment	Insulin injection	195	65.4
	Oral antihyperglycemic	89	29.9
	Both	15	4.7
BMI(kg/m^2^)	Underweight (< 18.5)	50	16.7
	Normal weight1 (8.5–24.9)	198	66.2
	Overweight (25–29.9)	47	15.7
	Obese (30 and above)	4	1.3
Having glucometer	Yes	40	13.1
	No	259	86.9
Diabetic complications	Yes	85	25.1
	No	214	74.9
Presence of comorbidities	Yes	81	27.1
	No	218	72.9
Duration of diagnosis	< 5	103	34.4
	5–10	95	31.8
	> 10	101	33.8

*DM* diabetes mellitus, *BMI *body mass index

### Factors associated with diabetic self-care

Overall, 53.3% of the study participants had good diabetes self-care practice. Diabetic patients aged 25–34 years were 8.95 times (AOR: 8.95, 95% CI 1.89, 42.48) more likely to have good self-care practice than those who were above 65. The odds of good diabetic self-care practice were 2.5 times (AOR: 2.5, 95% CI 1.07, 5.85) higher among respondents who earned a high monthly income than those who earned lower monthly income.

Study participants who had a family history of DM were 4.5 times (AOR: 4.5, 95% CI 1.3, 15.5) more likely to practice self-care habits than those who had no family history of DM. Respondents who were diagnosed before 10 years were two times (AOR: 2.14, 95% CI 1.13,4.05) more likely to have self-care practice than those patients who had been less than 5 years after their DM diagnosis.

Those respondents who had no history of diabetic complications were 3.87 times (AOR: 3.87, 95% CI 2.002, 7.48) more likely to follow the recommended self-care practice than those patients who had diabetic complications. Study participants who had glucometer at their home were 4.077 times (AOR: 4.077, 95% CI 1.78, 9.33) to perform diabetic self-care practices than those who did not have the equipment at their home (Table 3).

**Table 3:** Factors associated with diabetic self-care practices among adults with diabetes mellitus on follow-up clinics at public hospitals of Arsi zone, southeast Ethiopia, 2017

Variables	Self-care practice status		COR (95% CI)	AOR (95%CI)	P value
	Poor N%	Good N%			
Age					
18–24	18 (12.95)	45 (28.13)	1.500 (0.593–3.791)*	10.44 (2.76–39.5)**	0.001
25–34	35 (25.2)	35 (21.9)	3.750 (1.423–9.883)*	8.95 (1.89–42.48)**	0.006
35–44	27 (19.42)	29 (18.13)	2.611 (0.619–4.193)*	7.24 (1.67–31.32)**	0.008
45–54	24 (17.3)	24 (15)	1.500 (0.563–3.997)	3.39 (0.75–15.27)	0.113
55–64	20 (14.4)	17 (10.63)	1.275 (0.456–3.567)	2.203 (0.48–10.16)	0.311
> 65	15 (10.8)	10 (6.25)	1	1	
Marital status					
Single	30 (21.6)	56 (35)	1.87 (1.198–2.908)	1.1 (0.49–2.40)	0.855
Married	104 (74.8)	98 (61.25)	.942 (.715–1.242)	1.3 (0.20–9.40)	0.824
Divorced	2 (1.44)	5 (3.13)	2.5 (.485–12.889)	0.3 (0.02–4.03)	0.347
Widowed	3 (2.16)	1 (0.625)	1	1	1
Monthly income					
Poor	71 (51.1)	87 (54.4)	1	1	
Medium	17 (12.2)	25 (15.6)	1.081 (0.569–2.054)	1.563 (0.839–2.912)	0.160
Rich	51 (36.7)	48 (30)	3.409 (1.684–6.898)*	2.495 (1.07–5.85)**	0.035
Family history of DM					
Yes	15 (10.79)	46 (28.75)	3.282 (1.737–6.201)*	4.49 (1.3–15.466)**	0.017
No	124 (89.21)	114 (71.25)	1	1	
Duration of diagnosis (years)					
< 5	52 (37.41)	50 (31.25)	1	1	
5–10	50 (35.97)	45 (28.13)	0.567 (0.324–0.992)	0.96 (0.511–1.801)	0.897
> 10	37 (26.62)	65 (40.62)	0.520 (0.294–0.921)*	2.14 (1.127–4.05)**	0.020
Diabetic complication					
Yes	32 (23)	53 (33.1)	1	1	
No	107 (77)	107 (66.9)	2.634 (1.502–4.621)*	3.87 (2.002–7.48)**	0.001
Presence of glucometer					
Yes	130(93.5)	129(80.6)	3.905(1.73–8.82)*	4.077(1.78–9.33)**	0.001
No	9(6.5)	31(19.4)	1	1	

*COR* crude odds ratio, *AOR* adjusted odds ratio, *CI* confidence interval

*P value < 0.05

**Shows statistically significant association

## Discussion

This study assessed self-care practice and its associated factors among adults with DM on follow up at Public hospitals of Arsi zone. Accordingly, 46.7% (95% CI 40.6–52%) of the respondents had poor self-care practices. This finding is almost in line with the studies conducted at Addis Ababa, Nekemte referral hospital, West Shoa, Benishangul Gumz Public Hospital, Hadiya Zone, Southwest Ethiopia, and the University of Gondar which were 44.4%, 45%, 45.5%, 45.7%, 47.7%, 50.8% and51.86% respectively [16–22].

On the other hand, the finding in the present study was higher than the studies carried out at Dilla referral hospital, Iran, Debre Tabor General Hospital, and west Ethiopia where only 23.2%, 26.2%, 36.9%, and 39.3% of the study participants had poor self-care practice respectively [23–26]. Lesser access to diabetes self-care-centered information in the present study setting, the difference in lifestyle and source of the population might be the reason for the variation since some of the previous studies included type 2 DM patients only.

On the contrary, the finding of this study was lower compared to the studies conducted in Kenya, Harari, Felegehiwot, and Ayder Comprehensive Specialized Hospitals where 59%, 60.8%, 63.2%, and 74.5% of the study participants had poor self-care practice respectively [27–30]. The variation might be the presence of cross-cultural habits and variation in lifestyle of the respondents. The difference in source population and study setting might have also part of the deviation of this finding. The study participants enrolled in the present study were patients with DM who had a follow-up in a diabetic clinic whereas the study which was conducted in Kenya was at a community setting which could result in poor self-care practice as lack of information on diabetes self-care practices that comes from the clinical setting.

This study revealed that the younger age group was 8.95 times more likely to have good self-care practice than those above 65. This finding was in line with the study conducted at Nekemt Referral Hospital and Egypt [17, 31]. The reason for this finding could be young age which gives chance to get a healthy diet and perform regular physical exercise easily than those who are aged.

The odds of good diabetic self-care practice were 2.5 times higher in respondents who earned high monthly income than those who earned low monthly income. This finding is comparable with the study conducted in Gondar, Ayder Comprehensive Specialized Hospital, and India [22, 30, 32]. This might be due to the fact that diabetes patients who earn a high monthly income could afford to get diets that are recommended for DM patients and can also buy glucometer to monitor self-blood glucose level.

On the other hand, study participants who had a family history of DM were 4.5 times more likely to practice self-care habits than those who had no family history of DM. This could be because of experience sharing among family members.

Respondents who had a long duration of diagnosis were two times more likely to have good self-care practice than those patients with a short duration of a diabetes diagnosis. This study is in line with the studies conducted in West Shoa, Iran, and west Ethiopia [18, 24, 26]. The best reason for good self-care practice in patients with a long duration of diagnosis could be due to more contact with health care providers that may benefit them to get regular counseling and help improve self-care practices.

Those respondents who had no diabetic complications were 3.87 times more likely to follow the recommended self-care practice than those patients who had diabetic complications. This finding was congruent with the study conducted at the University of Gondar Referral Hospital [22]. This could be due to the fact that patients who had not developed complications could care for themselves easily.

Study participants who had a glucometer at their home were 4.077 more likely to perform diabetic self-care practices than those who did not have a glucometer. The finding in this study is in line with the study conducted at Benishangul Gumz Public Hospitals, Hadiya zone, and Debre Tabor General Hospital [19, 20, 25]. The possible reason might be having a glucometer could give chance to have regular self-blood sugar measurement and result in better metabolic control.

## Conclusion

The finding of this study revealed that a substantial number (46.7%) of respondents had poor diabetic self-care practice. Efforts should be made in the prevention of complications and concerning bodies should support those patients who are, aged, earning low monthly income, and who do not have glucometer at their home. Particularly, health care providers should give special attention to newly diagnosed patients.

## Limitation

The use of self-report of the study participants about self-care practice than direct observation was the limitation of the study which could result in social desirability bias.

## Data Availability

To keep patients’confidentiality, the raw data would not be shared. But, it is available from the corresponding author on reasonable request.

## Abbreviations

AOR: Adjusted odds ratio
BMI: Body mass index
CI: Confidence interval
COR: Crude odds ratio
DM: Diabetes mellitus
Epi info: Statistical Package for epidemiological information analysis
FBS: Fasting blood sugar
IDF: International diabetes association
SDSCA: Summary of diabetic self-care activities
SPSS: Statistical package for social science
WHO: World Health Organization
ETB: Ethiopian Birr
